# Cortical potentiation induced by calcitonin gene-related peptide (CGRP) in the insular cortex of adult mice

**DOI:** 10.1186/s13041-020-00580-x

**Published:** 2020-03-09

**Authors:** Yinglu Liu, Qi-Yu Chen, Jung Hyun Lee, Xu-Hui Li, Shengyuan Yu, Min Zhuo

**Affiliations:** 1 Center for Neuron and Disease, Frontier Institutes of Science and Technology, Xi’an Jiaotong University, Xi’an, China; 2 Department of Physiology, University of Toronto, 1 King’s College Circle, Toronto, Ontario M5S 1A8 Canada; 3 Medical School of Chinese PLA and Department of Neurology, The First Medical Centre, Chinese PLA General Hospital, Beijing, China; 4 Institute for Brain Research, QingDao International Academician Park, Qing Dao, China

**Keywords:** Calcitonin gene-related peptide, Insular cortex, Synaptic transmission, Adenylyl cyclase subtype 1

## Abstract

Recent studies demonstrate that calcitonin gene-related peptide (CGRP) plays critical roles in migraine. Immunohistochemistry and in situ hybridization studies have shown that CGRP and its receptors are expressed in cortical areas that are critical for pain perception including the anterior cingulate cortex (ACC) and insular cortex (IC). Recent studies reported that CGRP enhanced excitatory transmission in the ACC. However, little is known about the possible effect of CGRP on excitatory transmission in the IC. In the present study, we investigated the role of CGRP on synaptic transmission in the IC slices of adult male mice. Bath application of CGRP produced dose-dependent potentiation of evoked excitatory postsynaptic currents (eEPSCs). This potentiation was NMDA receptor (NMDAR) independent. After application of CGRP1 receptor antagonist CGRP_8–37_ or BIBN 4096, CGRP produced potentiation was significantly reduced. Paired-pulse facilitation was significantly decreased by CGRP, suggesting possible presynaptic mechanisms. Consistently, bath application of CGRP significantly increased the frequency of spontaneous and miniature excitatory postsynaptic currents (sEPSCs and mEPSCs). By contrast, amplitudes of sEPSCs and mEPSCs were not significantly affected. Finally, adenylyl cyclase subtype 1 (AC1) and protein kinase A (PKA) are critical for CGRP-produced potentiation, since both selective AC1 inhibitor NB001 and the PKA inhibitor KT5720 completely blocked the potentiation. Our results provide direct evidence that CGRP contributes to synaptic potentiation in the IC, and the AC1 inhibitor NB001 may be beneficial for the treatment of migraine in the future.

## Introduction

The neuropeptide of calcitonin gene-related peptide (CGRP) is a 37–amino acid peptide that is a member of the calcitonin family. It is widely expressed in the central and peripheral nervous systems, and frequently coexists and interacts with other neurotransmitters [1]. As a multifunctional neuropeptide, CGRP exists in two distinct isoforms: α-CGRP (CGRP1), which is the product of alternative splicing of the calcitonin gene in neurons, and β-CGRP (CGRP2), which is encoded by a separate gene [2]. CGRP1 is the dominant type of distribution in the central nervous system (CNS), and is detected in nearly 50% of the neurons in the trigeminal vascular system [3, 4]. CGRP2 is particularly prominent in the enteric nervous system [5]. It is known the trigeminal vascular system is highly related with pain, especially the migraine [6]. Many studies show that CGRP reliably released by the activation of primary sensory neurons in the trigeminal vascular system during migraine attacks and the plasma level of CGRP could increase in ictal as well as interictal periods among migraineurs [7]. Intravenous administration of CGRP could induce migraine and Erenumab, a human monoclonal antibody blocking the CGRP receptor, is found to be valid for clinical treatment of migraine [8, 9]. It is likely that antibodies produce effect through peripheral mechanisms, since they are poor to permeate blood-brain barrier [10].

Insular cortex (IC), as an integrating forebrain structure, is believed to be involved in pain perception as well as other higher brain functions such as emotional and cognitive functions [11–18]. For example, Qiu et al. demonstrate that the excitatory transmission in the IC was enhanced after peripheral nerve injury [15], and Liu et al. provide the first in vitro report of long-term potentiation (LTP) in the IC using 64-channel recording system [11]. Neurons in the IC may integrate extero- and interoceptive information, and such process may plays a vital role as “cortical hub” in migraine attacks. Human imaging studies have found that the IC is activated in migraineurs [19, 20]. Cumulative evidences suggest that CGRP-containing pathways could convey nociceptive and visceral sensation from the posterior thalamus and parabrachial nuclear (PBN) complex to the IC and the amygdala [2]. Immunohistochemistry and in situ hybridization researches have also shown that CGRP and its receptors are expressed in some cortical areas, including the IC and anterior cingulate cortex (ACC) [21, 22]. Our previous study indicates that CGRP may contribute to synaptic potentiation in the ACC [21]. The ACC and IC are two major cortical areas for pain perception [23–26], less information is available about the effects of CGRP on excitatory transmission within the IC. In the present study, we used whole-cell patch-clamp recording of adult mice slices to investigate the effects of CGRP on synaptic transmission in the IC. We found that bath application of CGRP produced dose-dependent potentiation, which was the NMDA receptor-independent. Application of CGRP1 receptor antagonist attenuated this potentiation. Bath application of CGRP significantly increased the frequency of spontaneous and miniature excitatory postsynaptic currents (sEPSCs and mEPSCs) and was consistent with the decrease of paired-pulse ratio (PPR) found in evoked excitatory postsynaptic currents (eEPSCs), which suggested that CGRP enhanced the glutamate release from the presynaptic terminals. Finally, both selective AC1 inhibitor NB001 and the PKA inhibitor KT5720 completely blocked the potentiation, demonstrating that calcium-stimulated cAMP pathway was critical for CGRP-produced potentiation in the IC.

## Methods

### Animals

Adult male C57BL/6 mice (7–9 weeks old) were used. All animals were housed under a 12 h light/dark cycle with the food and water provided ad libitum. Experiments were conducted under the protocol approved by the Animal Care and Use Committee of the University of Toronto (Protocol ID, 20012315).

### Brain slices preparation

Coronal brain slices (300 μM) at the level of the IC were prepared using standard methods [27–29]. Briefly, mice were deeply anesthetized with 5% isoflurane and sacrificed by decapitation. The whole brain was removed quickly from the skull and submerged in the oxygenated (95% O_2_ and 5% CO_2_) ice-cold artificial cerebrospinal fluid (ACSF) containing (in mM) 124 NaCl, 2.5 KCl, 2 MgSO4, 1 NaH_2_PO4, 2 CaCl_2_, 25 NaHCO_3_, and 10 D-glucose. The whole brain tissue was cooled for short time before trimmed as the proper part to glue onto the microslicer (VT1200S Vibratome, Leica, Germany). Slices were incubated in a submerged recovery chamber at room temperature for 1 h. The ACSF were continuously aerated with a mixture of 95% O_2_ and 5% CO_2_.

### Whole-cell patch-clamp recording

Whole cell recordings were performed in a recording chamber on the stage of an Axioskop 2FS microscope with infrared differential interference contrast optics for visualization. eEPSCs were recorded from layer II/III neurons with an Axon 200B amplifier (Molecular Devices), and the stimulations were evoked in layer V of the IC by a bipolar tungsten stimulating electrode. The recording pipettes (3–5 MΩ) were filled with the solution containing (in mM) 145 K-gluconate, 5 NaCl, 1 MgCl_2_, 0.2 EGTA, 10 HEPES, 2 Mg-ATP, and 0.1 Na_3_-GTP, which adjusted to pH 7.3 with KOH and had osmolality of 300 mOsmol. The amplitude of eEPSCs were adjusted between 100 to 150 pA to obtain a baseline. For mEPSCs recording, tetrodotoxin (TTX, 1 μM) was added in the perfusion solution. Picrotoxin (PTX, 100 μM) was always presented to block the GABA_A_ receptor-mediated inhibitory synaptic currents in all experiments. Access resistance was 15–30 MΩ and monitored throughout the experiment. Data were discarded if access resistance changed > 15% during an experiment. Data were filtered at 1 kHz, and digitized at 10 kHz.

### Drugs

The chemicals and drugs used in this study were as follows: α-CGRP and CGRP_8–37_ were obtained from BACHEM AG (Bubendorf, Switzerland). BIBN 4096 were purchased from Tocris Cookson (Bristol, UK) and PTX was bought from Sigma-Aldrich (St Louis, MO, USA). All the other drugs were purchased from HelloBio (Princeton, NJ, USA) except NB001, which was provided by NeoBrain Pharmac Inc. (Canada). Drugs were prepared as stock solutions for frozen aliquots at − 20 °C. All these drugs were diluted from the stock solution to the final desired concentration in the ACSF before being applied to the perfusion solution.

### Statistical analysis

Whole-cell patch-clamp data were collected and analyzed by Clampex 9.0 and Clampfit 9.0 software (Molecular Devices). For the eEPSCs, the amplitude was normalized and expressed as the percentage of the baseline. Spontaneous and miniature EPSCs were analyzed by an event detection program (Mini Analysis Program; Synaptosoft, Inc., Decatur, GA). The paired *t* tests or one-way ANOVA was conducted as appropriate. The *Tukey* test was used for post hoc comparison. GraphPad Prism 7.0 software (GraphPad Software, San Diego, CA) and SPSS version 22.0 (SAS Institute Inc., Cary, NC) software were used plotting figures and analyzing results. All data were presented as the mean ± standard error of the mean (SEM). In all cases, *p* < 0.05 was considered statistically significant.

## Results

### CGRP enhanced excitatory synaptic transmission in the IC

**Fig. 1:**
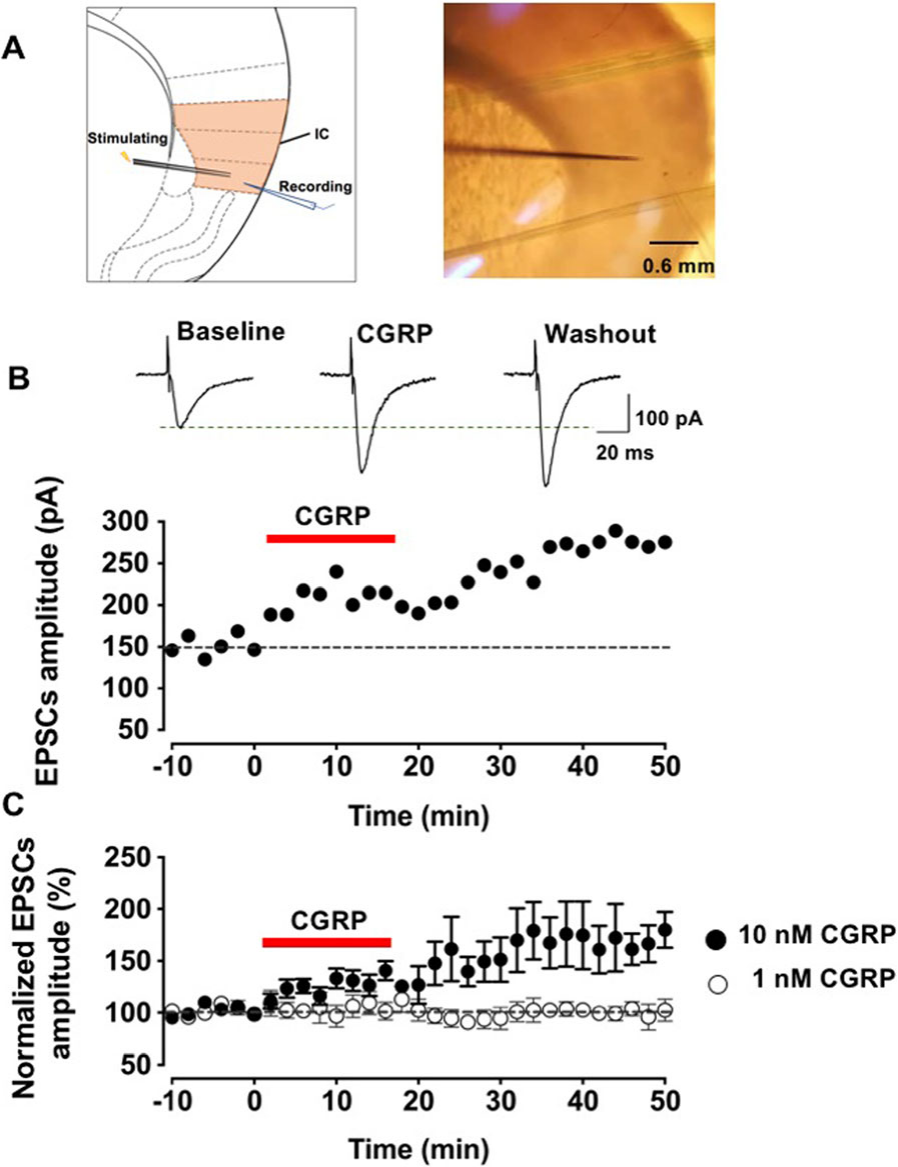
CGRP (10 nM) enhanced excitatory synaptic transmission in the IC. **a** Schematic diagram (left) and representative recording diagram (right) showed the placement of stimulating and recording electrodes in the IC. **b** Top: sample traces of EPSCs with single-pulse stimulation during the baseline, the application of CGRP and washout period. Bottom: a time course plot of a representative single example. **c** The averaged data showed the amplitude significantly increased during the application of CGRP (10 nM) and washout period when compared with baseline. The 1 nM CGRP were used as a control, indicating the dose-dependent characteristic of the effect of CGRP (*n* = 9 neurons/6 mice for 10 nM CGRP, filled circles; *n* = 6 neurons/4 mice for 1 nM CGRP, open circles)

Our recent study showed that CGRP increased the excitatory synaptic transmission in the ACC [21]. Here we wanted to see if similar effect would be found in the IC, which also plays important roles in pain perception and chronic pain. We recorded EPSCs at pyramidal neurons of layer II/III in the agranular and dysgranular insular cortex in the presence of a GABA_A_ receptor antagonist, PTX (100 μM). The holding potential was − 70 mV and local stimulation was given at layer V in the IC. The schematic diagram and representative recording diagram were shown as Fig. 1a. After achieving the stable baseline recording in response to single-pulse stimulation for at least 10 min, the CGRP (10 nM) were applied. As shown in Fig. 1b and c, amplitudes of EPSCs were significantly increased after applied the 10 nM CGRP (131.6 ± 10.3% of baseline, *p* < 0.01 as compared with baseline, one-way ANOVA, *n* = 9 neurons/6 mice). The potentiation induced by CGRP was long-lasting, and persisted during the washout period for at least 30 min (168.8 ± 23.1% of baseline, *p* < 0.01 compared with baseline, one-way ANOVA, *n* = 9 neurons/6 mice, filled circles, Fig. 1c). The effect of CGRP was dose-related. At a dose of 1 nM, CGRP failed to induce any significant potentiation (106.6 ± 10.8% of baseline, F _(2, 27)_ = 2.4, *p* = 0.1, one-way ANOVA, *n* = 6 neurons/4 mice, open circles, Fig. 1c). Furthermore, there was no further potentiation after the washout of CGRP (102.1 ± 7.1% of baseline, F _(2, 27)_ = 2.4, *p* = 0.1, one-way ANOVA, *n* = 6 neurons/4 mice, open circles, Fig. 1c).

### The potentiation induced by CGRP was NMDAR independent

**Fig. 2:**
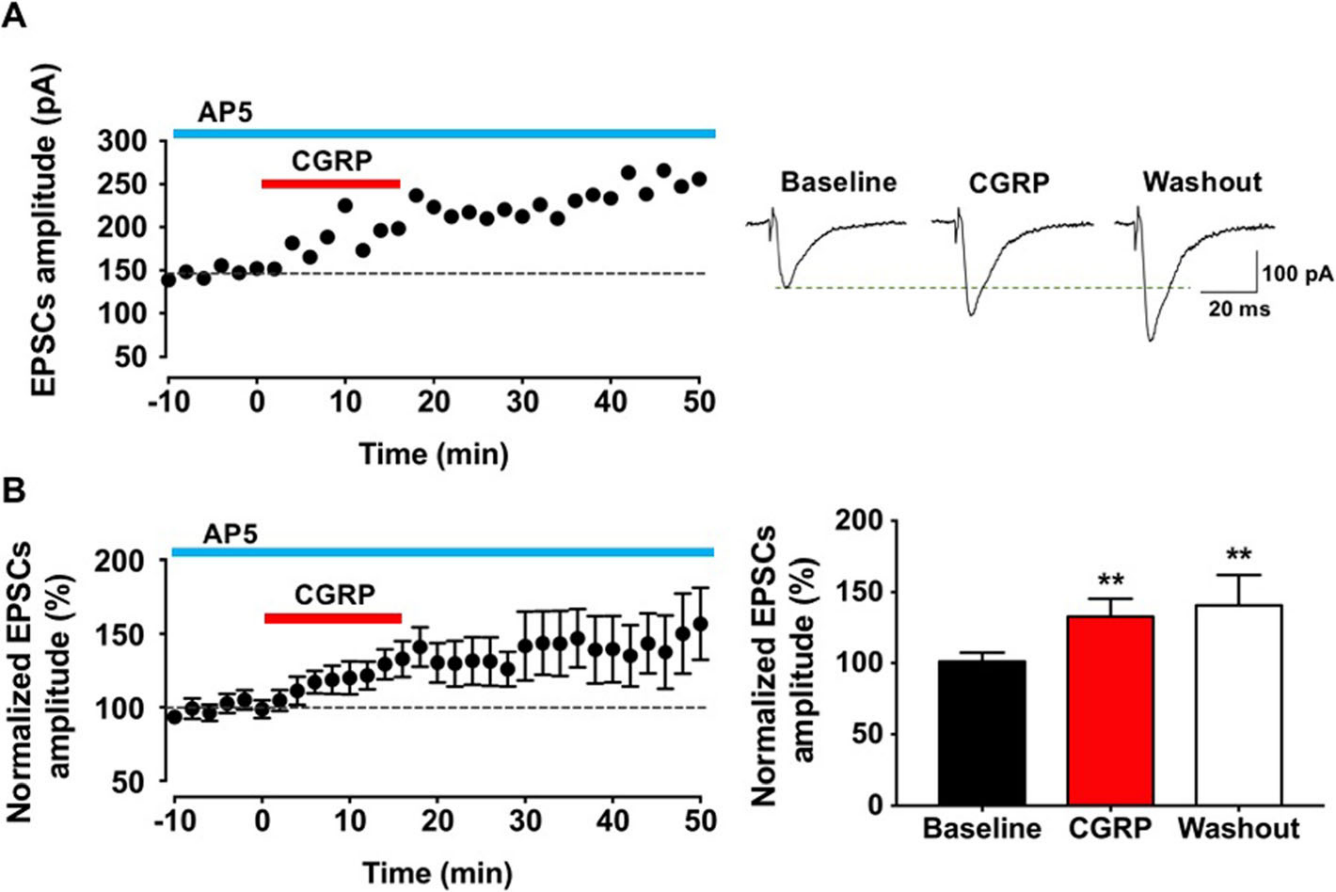
The potentiation induced by CGRP was NMDA receptor independent. **a** An NMDA receptor antagonist, AP5 (50 μM), did not affect the CGRP induced potentiation. Left: a time line plot of one representative sample. Right: sample traces showed the increased of the amplitude during the application of CGRP (10 nM) and washout period. **b** Left: the pooled data illustrated the time course of the effect of CGRP (10 nM) in the IC. Right: summarized data showed the amplitude significantly increased during the application of CGRP and washout period when compared with baseline (*n* = 8 neurons/5 mice). **p < 0.01 compared with baseline, error bars indicated SEM

Since the activation of NMDARs is crucial for most forms of LTP, we would like to test if CGRP induced potentiation is NMDAR dependent. In this set of experiments, an NMDAR antagonist, AP5 (50 μM) was present in the bath solution throughout the experiments. Bath application of CGRP caused similar amount of potentiation as those without AP5 (see above) (CGRP: 132.9 ± 12.5% of baseline, washout: 140.7 ± 21.3% of baseline, *p* < 0.01 and *p* < 0.01 compared with baseline, respectively; one-way ANOVA, *n* = 8 neurons/5 mice, Fig. 2).

### CGRP1 receptor was involved in the potentiation induced by CGRP

**Fig. 3:**
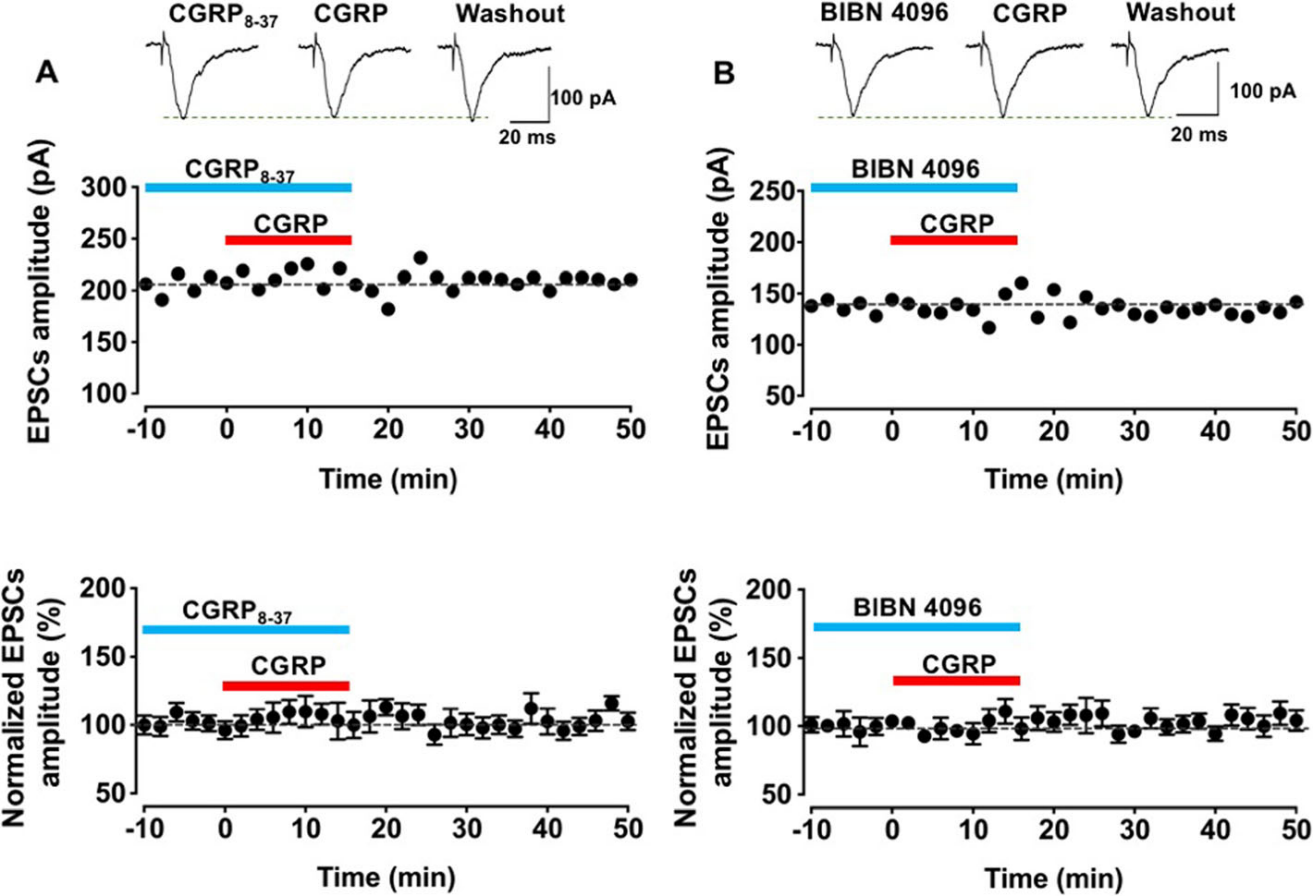
CGRP1 receptor was involved in the potentiation induced by CGRP. The peptide CGRP1 receptor antagonist CGRP_8–37_ (1 μM) (**a**) and non-peptide CGRP1 receptor antagonist BIBN 4096 (1 μM) (**b**) were applied during the baseline and added CGRP periods. Top: sample traces showed the amplitude of the baseline, application of CGRP (10 nM) and washout period for CGRP_8–37_ (1 μM) (**a**) and BIBN 4096 (1 μM) (**b**). Middle: a time line plot of one representative sample for CGRP_8–37_ (1 μM) (**a**) and BIBN 4096 (1 μM) (**b**). Bottom: Pooled data showed no differences among baseline, applied CGRP and washout periods for CGRP_8–37_ (1 μM, *n* = 7 neurons/5 mice) (**a**) and BIBN 4096 (1 μM, *n* = 8 neurons/4 mice) (**b**)

Among several receptors for CGRP, CGRP1 receptor is the dominant type that distributes in the CNS [3, 4]. We next tested the role of CGRP1 receptor in this CGRP induced potentiation. Two different antagonists, peptide CGRP_8–37_ and non-peptide antagonist BIBN 4096, were used. As shown in Fig. 3, after application of CGRP_8–37_ or BIBN 4096, 10 nM CGRP produced potentiation was significantly reduced (CGRP_8–37_: F _(2, 27)_ = 0.3, *p* = 0.7, one-way ANOVA, *n* = 7 neurons/5 mice, Fig. 3a; BIBN 4096: F _(2, 27)_ = 0.5, *p* = 0.6, one-way ANOVA, *n* = 8 neurons/4 mice, Fig. 3b). These results indicated that CGRP1 mediated CGRP induced potentiation in the IC.

### Effects on paired-pulse ratio by CGRP

**Fig. 4:**
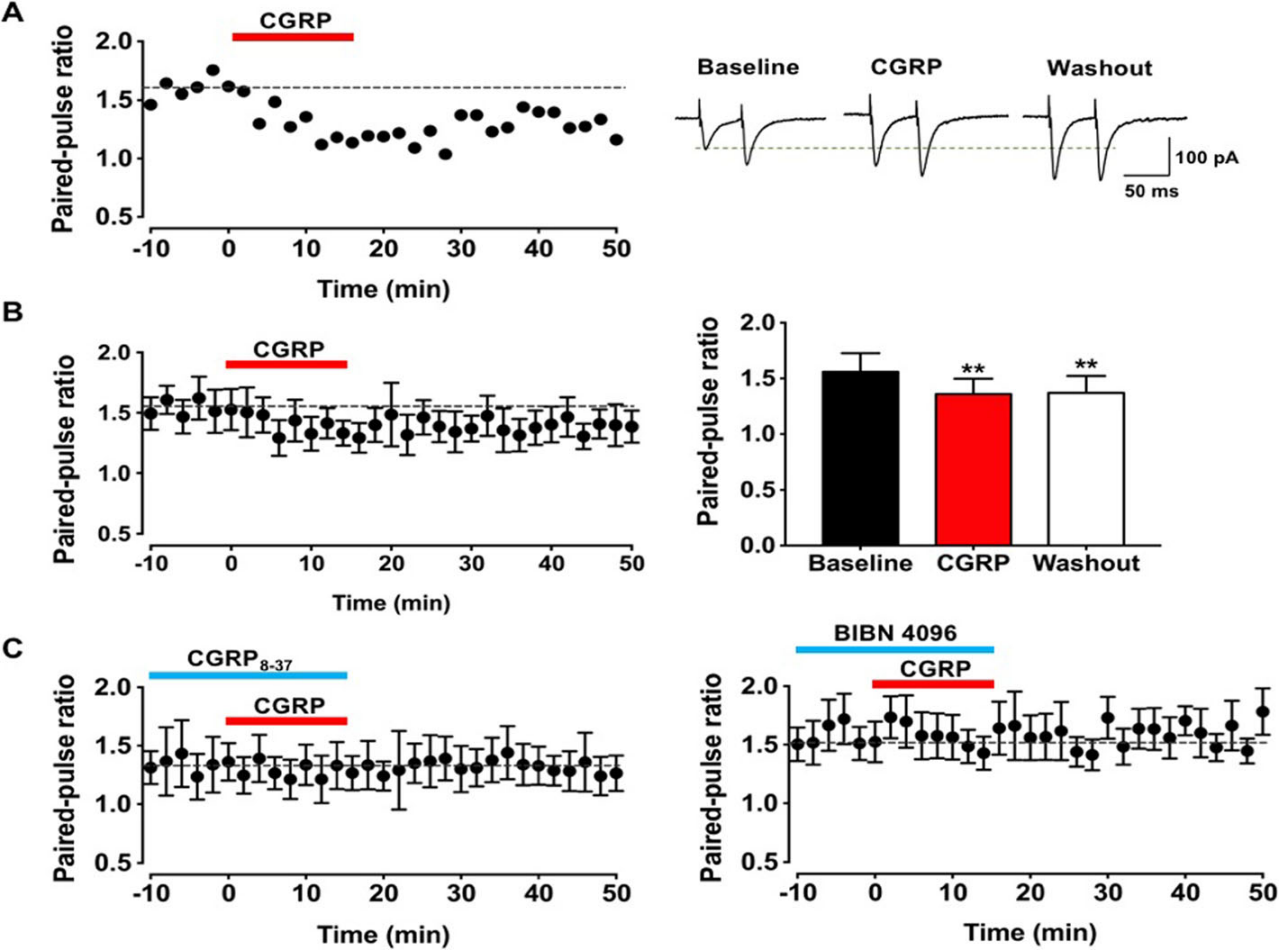
CGRP altered paired-pulse ratio in the IC. **a** Left: a time line plot of one representative sample. Right: sample traces of this neuron. **b** The time courses plot (left) and histograms (right) for the pooled data illustrated that PPR significantly decreased during the application of CGRP (10 nM) and washout period when compared with baseline (*n* = 9 neurons/6 mice). **c** CGRP1 receptor antagonist CGRP_8–37_ (left) and non-peptide CGRP1 receptor antagonist BIBN 4096 (right) blocked the decrease of PPR after applied CGRP (CGRP_8–37_, *n* = 7 neurons/5 mice; BIBN 4096, *n* = 8 neurons/4 mice). **p < 0.01 compared with baseline, error bars indicated SEM

Next, we would like to determine if CGRP may produce potentiation by enhancing the release of transmitters. Paired-pulse responses to a paired stimulation at 50 ms interval were collected. We calculated PPR before and after CGRP application. As shown in Fig. 4a and b, sample traces and pooled data showed that PPR was significantly reduced after applied CGRP and the reduction was long-lasting during the washout period (baseline: 1.6 ± 0.2, CGRP: 1.4 ± 0.1, washout: 1.4 ± 0.2, *p* < 0.001 and *p* < 0.001 compared with baseline, respectively; one-way ANOVA, *n* = 9 neurons/6 mice). Furthermore, in experiments with the CGRP_8–37_ or BIBN 4096, the reduction of PPR were blocked (CGRP_8–37_: F _(2, 27)_ = 0.5, *p* = 0.6, one-way ANOVA, *n* = 7 neurons/5 mice, Fig. 4c left; BIBN 4096: F _(2, 27)_ = 0.6, *p* = 0.5, one-way ANOVA, *n* = 8 neurons/4 mice, Fig. 4c right). These data suggested that CGRP may produce its effect by affecting the release of glutamate, although we cannot completely rule out possible postsynaptic effects as well.

### Effect of CGRP on sEPSCs

**Fig. 5:**
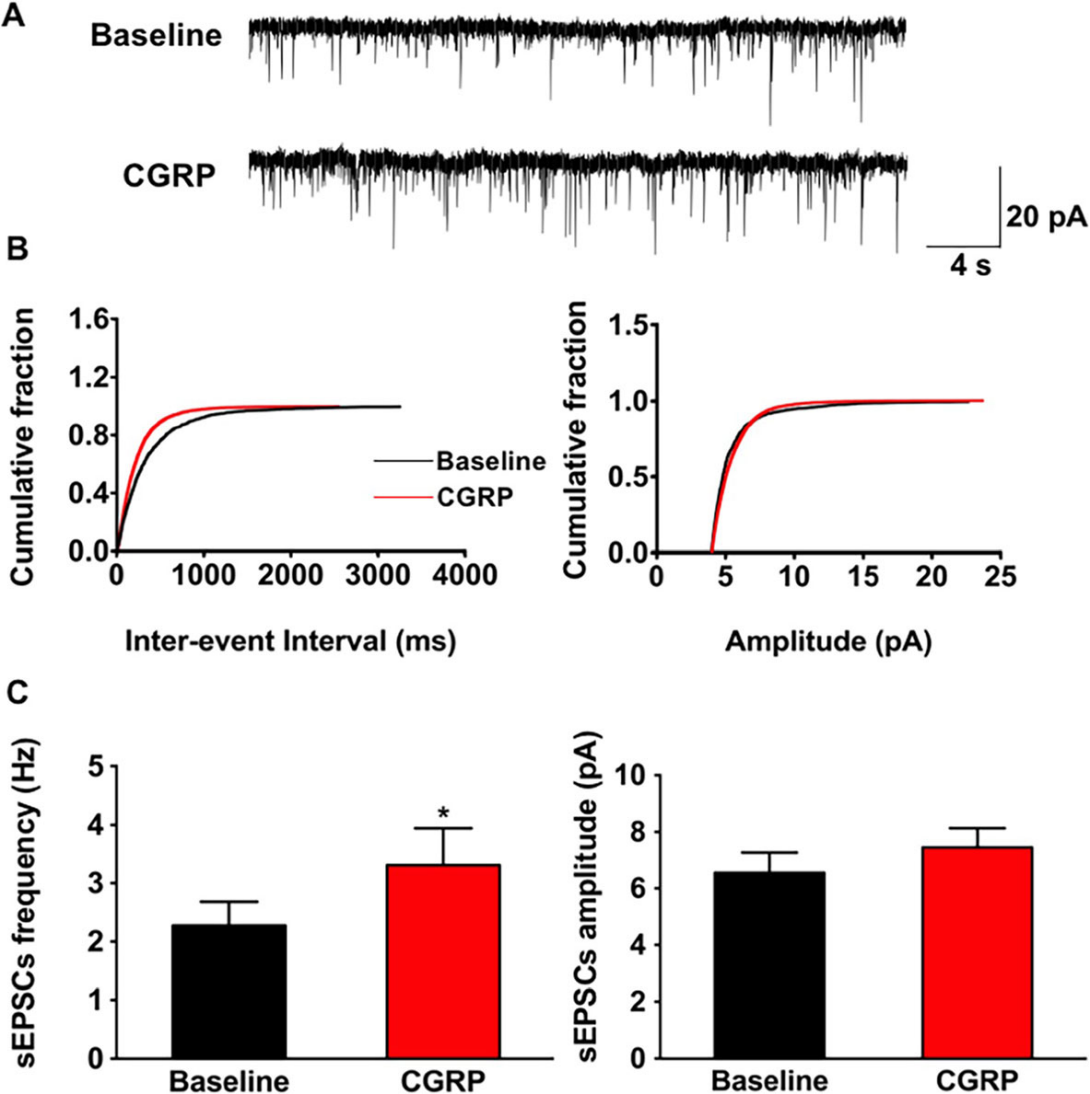
CGRP increased the frequency of sEPSCs. **a** Representative traces of the sEPSCs recorded in the IC neurons before and after applied CGRP (10 nM). **b** Cumulative fraction of inter-event interval (left) and amplitude (right) of the sEPSCs in the phase of baseline (black line) and CGRP application (red line). **c** Statistic results of the frequency (left) and amplitude (right) of sEPSCs (*n* = 9 neurons/6 mice). *p < 0.05, error bars indicated SEM

Spontaneous events are thought to be the results of the presynaptic action potential evoked neurotransmitter vesicles release from the readily releasable pool [30]. The effects of CGRP on sEPSCs recorded from the pyramidal neurons of the IC were examined. As shown in Fig. 5, the frequency of sEPSCs was significantly increased with the bath application of 10 nM CGRP (2.3 ± 0.4 Hz vs. 3.3 ± 0.6 Hz, t = − 2.8, *p* < 0.05, paired *t* test, *n* = 9 neurons/6 mice). A cumulative fraction plot showed that the inter-event interval was reduced during CGRP application (Fig. 5b left). While, the amplitude of sEPSCs was not significantly affected (6.6 ± 0.7 pA vs. 7.5 ± 0.7 pA, t = − 1.3, *p* = 0.2, paired *t* test, *n* = 9 neurons/6 mice, Fig. 5b and c right). Next, we examined if the effects of CGRP can be blocked by CGRP1 receptor antagonists. We found that both CGRP_8–37_ and BIBN 4096 blocked the effects of CGRP on sEPSCs (CGRP_8–37_: frequency: 3.0 ± 0.5 Hz vs. 3.1 ± 0.6 Hz, t = − 0.6, *p* = 0.5, paired *t* test, *n* = 7 neurons/4 mice; amplitude: 10.9 ± 1.0 pA vs. 10.7 ± 0.8 pA; t = 0.7, *p* = 0.5, paired *t* test, *n* = 7 neurons/4 mice; BIBN 4096: frequency: 2.9 ± 0.4 Hz vs. 3.1 ± 0.4 Hz, t = − 1.9, *p* = 0.1, paired *t* test, *n* = 8 neurons/4 mice; amplitude: 10.0 ± 1.0 pA vs. 10.1 ± 1.0 pA, t = − 1.3, *p* = 0.2, paired *t* test, *n* = 8 neurons/4 mice). These results suggested that CGRP effects maybe mainly presynaptic and needs CGRP1 receptors.

### Effect of CGRP on mEPSCs

**Fig. 6:**
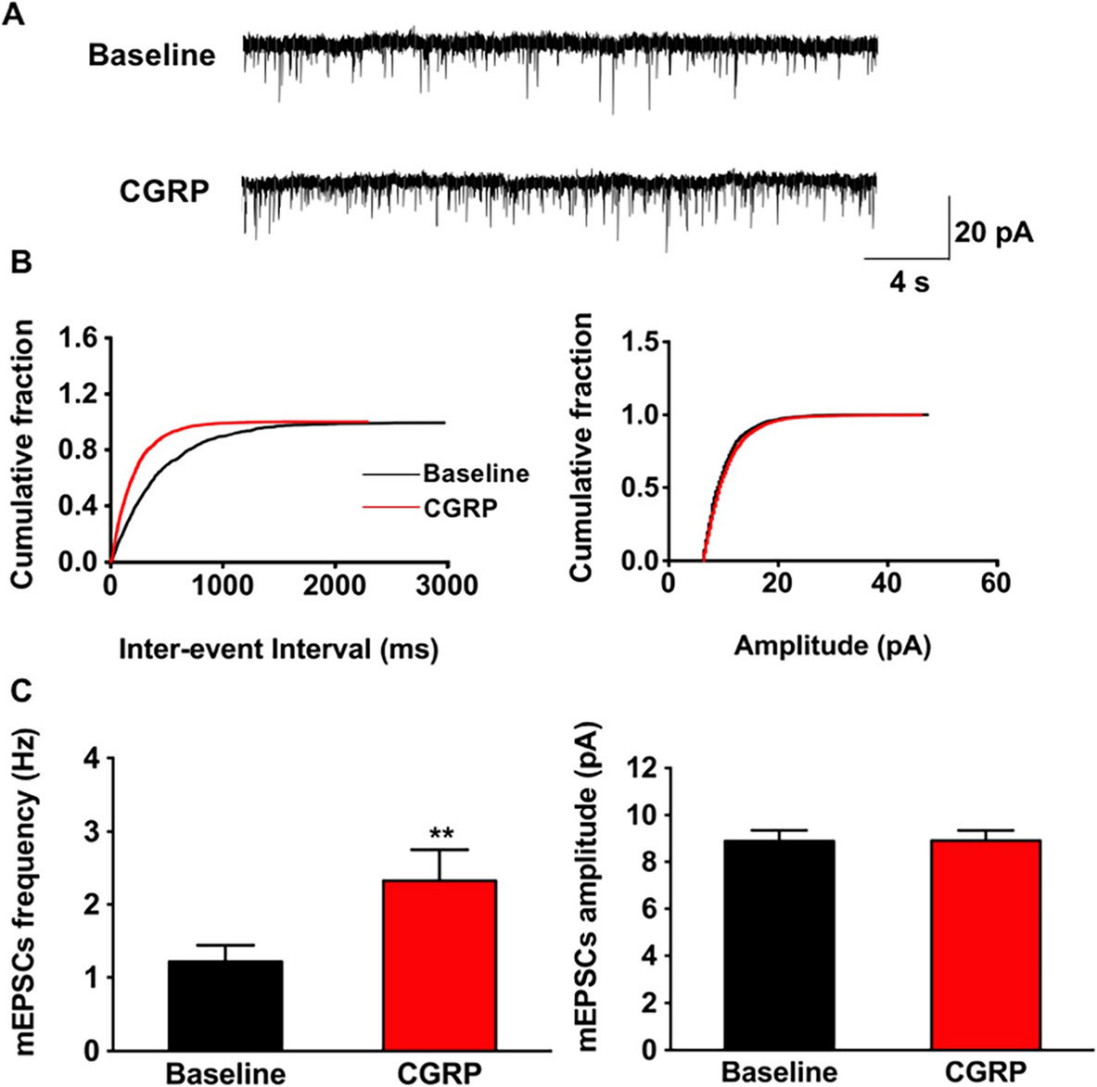
CGRP increased the frequency of mEPSCs. **a** Representative traces of the mEPSCs recorded in the IC neurons before and after applied CGRP (10 nM). **b** Cumulative fraction of inter-event interval (left) and amplitude (right) of the mEPSCs in the phase of baseline (black line) and CGRP application (red line). **c** Statistic results of the frequency (left) and amplitude (right) of mEPSCs (*n* = 9 neurons/5 mice). **p < 0.01, error bars indicated SEM

Unlike the sEPSCs, miniature synaptic transmission is resulted from neurotransmitter release independent of action potential, which occurs randomly in the absence of stimuli. We also recorded mEPSCs in the IC in the presence of 1 μM TTX to further determine the role of presynaptic mechanisms of CGRP induced potentiation. It found that the frequency of mEPSCs were significantly increased after perfusing 10 nM CGRP (1.2 ± 0.2 Hz vs. 2.3 ± 0.4 Hz, t = − 4.7, *p* < 0.01, paired *t* test, *n* = 9 neurons/5 mice, Fig. 6). Besides, a cumulative fraction plot showed a decrease of inter-event-interval during CGRP application (Fig. 6b). As to the amplitude of mEPSCs, no significant changes were observed (8.9 ± 0.4 pA vs. 8.9 ± 0.5 pA, t = 0.2, *p* = 0.8, paired *t* test, *n* = 9 neurons/5 mice, Fig. 6b and c right). We also found that the effect of CGRP on mEPSCs was blocked in the presence of CGRP_8–37_ or BIBN 4096 (CGRP_8–37_: frequency: 2.2 ± 0.3 Hz vs. 2.3 ± 0.3 Hz, t = − 1.1, *p* = 0.3, paired *t* test, *n* = 7 neurons/4 mice; amplitude: 8.8 ± 0.2 pA vs. 8.8 ± 0.1 pA, t = 0.3, *p* = 0.8, paired *t* test, *n* = 7 neurons/4 mice; BIBN 4096: frequency: 2.2 ± 0.4 Hz vs. 2.3 ± 0.5 Hz, t = − 1.4, *p* = 0.2, paired *t* test, *n* = 6 neurons/4 mice; amplitude: 9.7 ± 0.4 pA vs. 9.5 ± 0.4 pA, t = 1.9, *p* = 0.1, paired *t* test, *n* = 6 neurons/4 mice). These results demonstrated that CGRP enhanced excitatory synaptic transmission via increasing the probability of presynaptic neurotransmitter release in the IC and CGRP1 receptors are important for this process.

### AC1-PKA signal pathways were required for CGRP induced potentiation

**Fig. 7:**
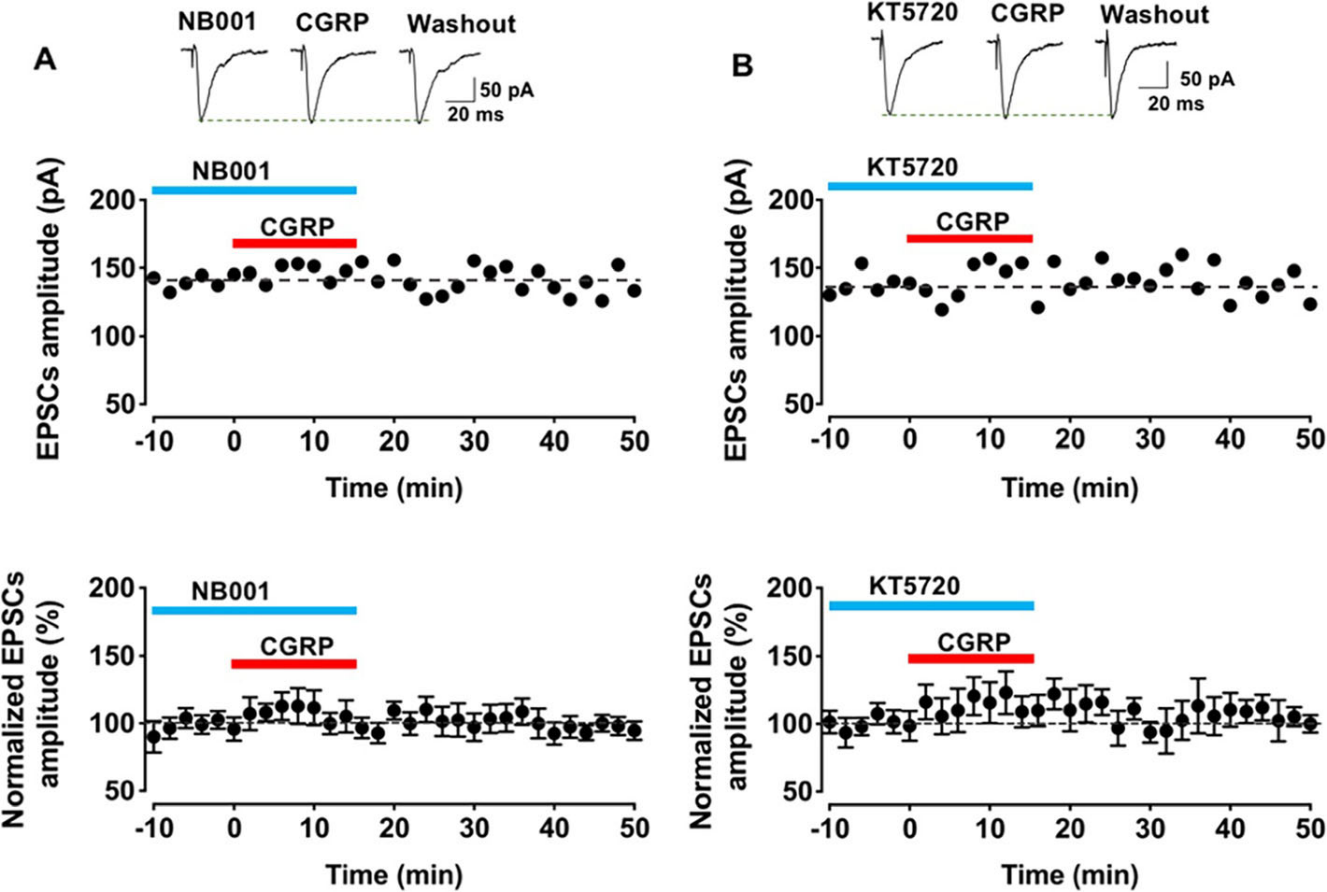
AC1-PKA signal pathways were involved in the CGRP induced potentiation. **a** Selective AC1 inhibitor, NB001 (50 μM) attenuated the effect of CGRP (10 nM) in the IC. Top: sample traces showed the amplitude of the baseline, application of CGRP (10 nM) and washout period for NB001 (50 μM). Middle: representative sample of EPSCs did not significantly change before and after added CGRP. Bottom: The averaged data did not show significant differences after applying CGRP (*n* = 7 neurons/5 mice). **b** PKA inhibitor, KT5720 (1 μM) inhibited the effect of CGRP (10 nM) in the IC. Top: Original traces showed the amplitude of the baseline, application of CGRP (10 nM) and washout period for KT5720 (1 μM). Middle: representative sample of EPSCs failed to show significant changes before and after added CGRP. Bottom: The averaged data did not find significant differences after applying CGRP (*n* = 7 neurons/4 mice)

The primary signal transduction pathway for the CGRP receptor is mediated by G*αs*, which activates AC, leading to the production of cyclic adenosine monophosphate (cAMP) and activation of protein kinase A (PKA) [2]. Consistently, our previous study showed that in the ACC, the CGRP induced potentiation did need AC1 and PKA [21]. Here we tried to determine if this signal pathway is also required in the IC. Firstly, a selective AC1 inhibitor, NB001 (50 μM) [31] was bathed during the baseline and CGRP periods. The results showed that NB001 completely blocked the effect of CGRP (F _(2, 27)_ = 0.2, *p* = 0.7, one-way ANOVA, *n* = 7 neurons/5 mice, Fig. 7a). Furthermore, a PKA inhibitor, KT5720 (1 μM) was found to attenuate CGRP produced effects (F _(2, 27)_ = 2.5, *p* = 0.1, one-way ANOVA, *n* = 7 neurons/4 mice, Fig. 7b). Our previous studies in the IC as well as ACC found that the same dose of inhibitor NB001 or KT5720 did not significantly affect baseline excitatory transmission [29, 31, 32].

## Discussion

**Fig. 8:**
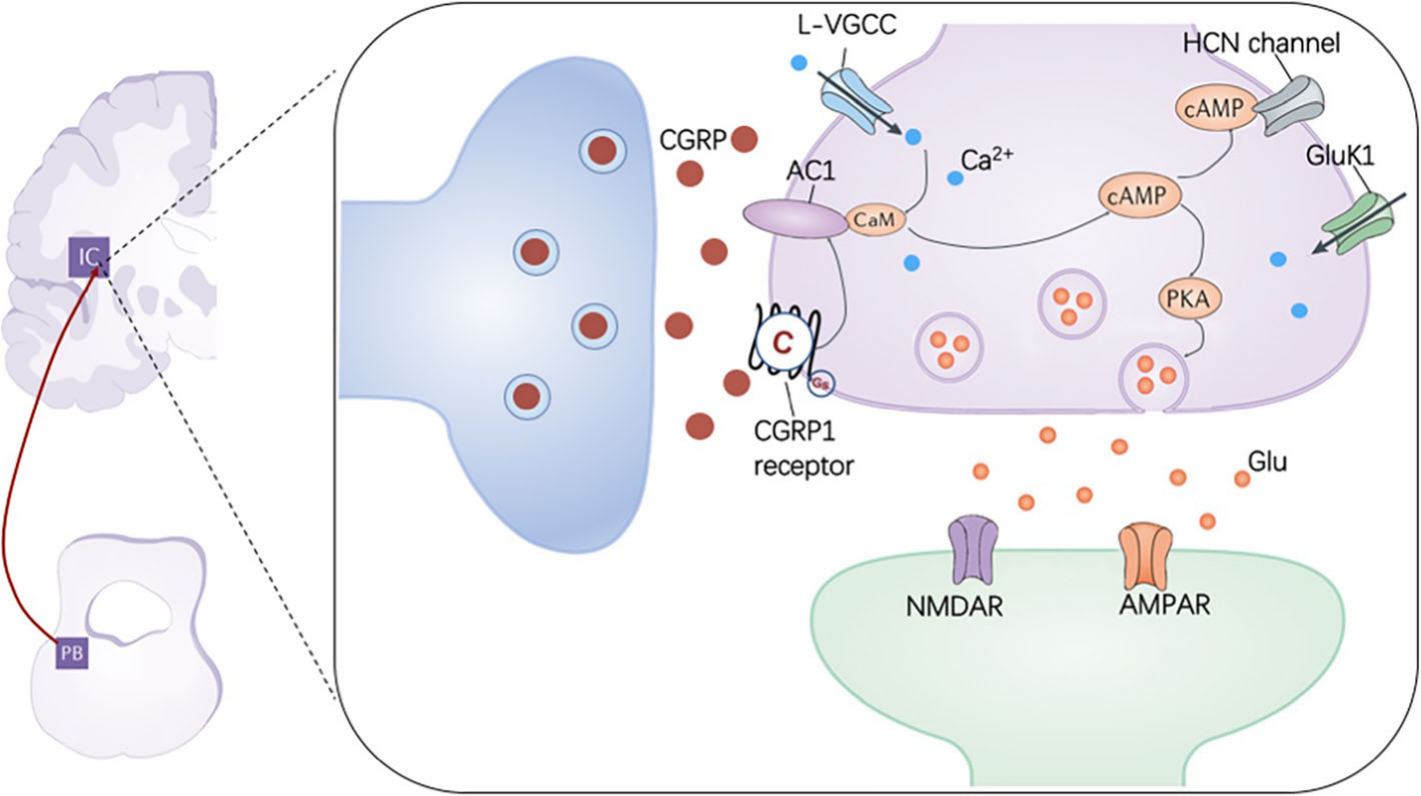
The proposed model for signaling pathways of CGRP induced potentiation. Left: a simplified diagram showed that the PBN is the prominent location of CGRP- expressing neurons in the CNS, which projects to the IC and other regions. Right: a synaptic model for the CGRP induced potentiation in the IC. CGRP likely causes the potentiation by enhancing presynaptic release of glutamate, although we cannot completely rule out the possibility of postsynaptic action. The calcium-stimulated AC1 is critical for CGRP induced potentiation and downstream protein kinase PKA is also required

CGRP is a recognized neuromodulator which released both at central and peripheral terminals of nociceptors. Accumulative evidence has shown that CGRP in the CNS can be a key modulator of pain via its involvement in brain circuits and may contribute to central sensitization [2, 4, 21, 33]. In the present study, we report that the modulatory effect of CGRP on synaptic transmission in the IC, a key cortical area for pain perception and chronic pain. The results show that CGRP induced potentiation in the IC is NMDA receptor independent. CGRP altered the PPR and increased the frequency of sEPSCs and mEPSCs in the IC. Our pharmacological data demonstrate that neuronal selective AC1 is critical for CGRP induced potentiation. Furthermore, downstream protein kinase PKA is also required (see Fig. 8 for a summarized model). This is the first time to report the requirement of AC1 for CGRP induced potentiation in the IC, raising the possibility that AC1 inhibitor may be beneficial for the treatment of CGRP related migraine.

### Innervation and distribution of CGRP in the IC

CGRP is known to be distributed in the CNS. At periphery, CGRP is expressed in a subgroup of small neurons in the dorsal root ganglion, trigeminal and vagal ganglia, which respond to various sensory stimuli including noxious stimuli. These neurons then project to the dorsal horn, trigeminal nucleus caudalis, or nucleus of the solitary tract. Within the CNS, the CGRP-containing pathways originated from the PBN and posterior thalamus convey nociceptive and visceral sensation to the amygdala and the IC [2]. The IC can be divided into three fields based on its cytoarchitecture and subcortical connections: the granular, dysgranular, and agranular insular areas. Previous study shows that modest numbers of CGRP-like immunoreactive fibers are distributed mainly in layers II, III, and V of the agranular and dysgranular IC with few are detected in the granular area [22]. Thus, we selected the layer II or III pyramidal neurons for recordings, and the effects induced by CGRP is likely to mimic those released from those projecting terminals within the IC.

### Modulation of synaptic transmission by CGRP in the IC

Previous studies of central synaptic transmission influenced by CGRP are mainly focused on the PBN, amygdala, the bed nucleus of the stria terminalis and spinal cords [17, 34, 35]. In the present study, we demonstrate that CGRP enhanced the synaptic transmission in the IC, which is like our recent study in the ACC [21]. We found that CGRP induced potentiation is NMDAR independent for the role of CGRP in the IC. Interestingly, in the amygdala, Okutsu et al. reported that CGRP enhanced NMDAR-mediated excitatory potentials [36]. It is likely that CGRP may affect excitatory synapses differently in various brain regions. Based on our current findings, CGRP likely causes the potentiation by enhancing presynaptic release of glutamate in the IC, but we cannot completely rule out the possibility of postsynaptic action of CGRP. Future studies are clearly needed. Among two different subtypes of CGRP receptors, CGRP1 receptor is mostly investigated. Less is known about CGRP2 receptor [5, 37]. Thus, we mainly focus on the CGRP1 receptor in the present study. We did find that CGRP1 receptors are involved in CGRP-induced potentiation. For signaling pathways, the results demonstrated that neuronal AC1 is important for CGRP produced presynaptic enhancement. Previous studies demonstrate that AC1 is cortical for both pre- and post-LTP in the ACC and IC [29, 32, 38]. These results further support the important roles of AC1 in presynaptic potentiation within cortical synapses.

### Functional and clinical implications

Recent studies have consistently demonstrated that cortical potentiation, including pre-LTP and post-LTP, play important roles in different types of chronic pain. The ACC and IC are two critical cortical areas that are involved in chronic pain and emotional changes [23, 29, 39]. Supporting these hypotheses, the present study and our recent study consistently show that CGRP produce long-lasting potentiation in the IC and ACC. Considering the important roles of CGRP in migraine, we believe that these cortical mechanisms may be at least in part involved in CGRP-related migraine [11–13, 15, 21]. The latest Global Burden of Disease (GBD) study reported that migraine takes the second place based on the GBD’s disease hierarchy and in the age of 15–49 years group, migraine is the top cause of years lived with disability [40, 41]. The estimated one-year prevalence of migraine is 9.3% in the mainland China, and the total estimated annual cost is USD 47.8 billion [42]. In addition to CGRP receptor antagonists that are currently used for the treatment of migraine, our results provide direct evidence that NB001, a selective AC1 inhibitor, completely occluded the enhanced effect of CGRP in the IC. We propose that NB001 which has been proved to be safe in both animals and humans, may be a promising novel drug for treating migraine in the future.

## Data Availability

The datasets used and/or analysed during the current study are available from the corresponding author on reasonable request.

## Abbreviations

AC: Adenylyl cyclase
ACC: Anterior cingulate cortex
ACSF: Artificial cerebrospinal fluid
AP5: D-2-amino-5-phosphonopentanoic acid
cAMP: Cyclic adenosine monophosphate
CGRP: Calcitonin gene-related peptide
CNS: Central nervous system
eEPSCs: Evoked excitatory postsynaptic currents
GBD: Global Burden of Disease
IC: Insular cortex
LTP: Long-term potentiation
mEPSCs: Miniature excitatory postsynaptic currents
NMDA: N-Methyl-d-aspartic acid
PBN: Parabrachial nuclear
PKA: Activation of protein kinase A
PPR: Paired-pulse ratio
PTX: Picrotoxin
SEM: Standard error of the mean
sEPSCs: Spontaneous excitatory postsynaptic currents
TTX: Tetrodotoxin
